# Traumatic spinal spondyloptosis presenting in a tertiary care unit in central Nepal

**DOI:** 10.12688/f1000research.133377.1

**Published:** 2023-05-09

**Authors:** Sunil Munakomi

**Affiliations:** 1 Neurosurgery, College of Medical Sciences, Chitwan, Nepal

**Keywords:** spondyloptosis, presentation, management, outcome

## Abstract

Introduction: Traumatic spinal spondyloptosis, though rare, harbingers a high risk of mortality as well as permanent and disabling neurological deficits. They invariably become socially aloof and marginalized in most parts of our subcontinent owing to the lack of dedicated rehabilitation units amid their poor economic status. There is a paucity of studies pertaining to such rare epiphenomenon within our region.

Materials ad Methods: A study of 16 patients presenting with spinal spondyloptosis from January 2017 to January 2023 in a tertiary care center in Nepal was undertaken. The clinical records of the patients were retrieved from the hospital record section to study the demographic variables, modes of injury, American Spinal Injury Association (ASIA) grades, salient radiological characteristics, management strategies, and the resultant clinical outcomes.

Result: The mean age of the cohorts in our study was 40 years with an age range of 25-80 years. Most of the patients presented in ASIA ‘A’ neurological grade (75%). The cervical spine was involved in the majority (68.75%) of cases. 8 (50%) patients left against medical advice, 2 (12.5%) were managed conservatively, and 6 (37.5%) were operated. The posterior-only approach was undertaken in 4(66.67%) cases. Tracheo-oesophageal fistula occurred in 2 (33.33%) patients. And cerebrospinal fluid (CSF) leak occurred in 2 (33.33%) patients. The overall hospital mortality was 3(37.5%). All the surviving patients were of the ASIA ‘E’ grades.

Conclusion: There is a continuum of physical, economic, psychological, and social burdens to both the patients and their care providers. Rehabilitation is the ‘bottleneck’ variable governing poor outcomes in our subcontinents. The poor economic status of the people has a ripple effect upon the same. This should also aid in the patient counseling as well as fostering the notion of the paramount need for dedicated neuro-rehabilitation units in our regions.

## Introduction

Traumatic spinal spondyloptosis is a rare entity compromising only 1% of all traumatic spinal injuries. ^1^ There is more than 100% subluxation between the adjacent vertebral bodies. ^2^ This harbingers a high risk of mortality as well as permanent and disabling neurological deficits among the survivors owing to complete cord transection. ^2^ The survivors are compelled to be dependent on others for lifelong even for carrying out their activities of daily living (ADLs). They invariably become socially aloof and marginalized in most parts of our subcontinent owing to the lack of dedicated rehabilitation units amid their poor economic status. ^2^ Since the majority of these cohorts present with poor American Spinal Injury Association (ASIA) neurological grades, the surgical dictum mostly involves anatomical fixation to assist in their early rehabilitative strategies. The 360 degrees global (combined anterior and posterior approaches) are recommended only in rare circumstances for patients presenting with good ASIA neurological grades. ^2^^,^^3^

The largest case series pertaining to this entity in the current literature has a sample size of only 20 patients. ^2^ There is a paucity of studies pertaining to such rare epiphenomenon within our region. This study should provide insights to help frame the management algorithm among similar cohorts of patients. This will also aid in the process of patient counseling as well as foster the notion of the paramount need for dedicated neuro-rehabilitation units in our regions.

## Methods

**Figure 1.**
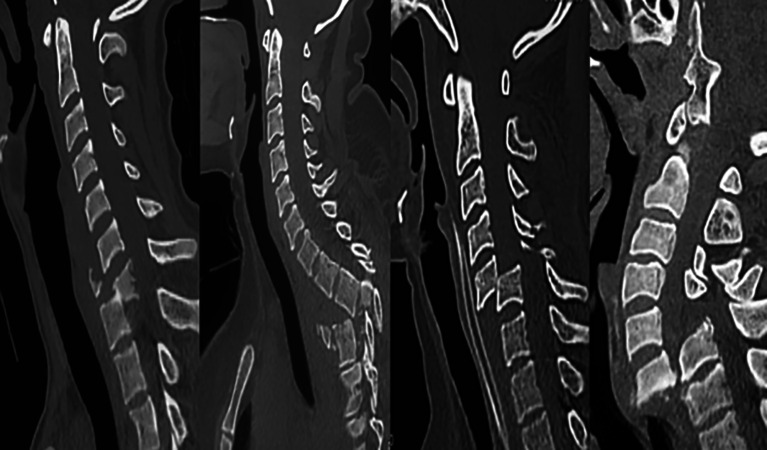
CT images of spondyloptosis at various anatomical levels of spine.

A descriptive study of consecutive cohorts of patients presenting with spinal spondyloptosis from January 2017 to January 2023 in the Emergency Department of the College of Medical Sciences was undertaken. Spondyloptosis was defined on radiological imaging (X-ray/CT/MRI) of the spine as >100% subluxation between the adjacent vertebra. The images of spondyloptosis at different levels of the spine have been demonstrated in Figure 1.

The clinical records of the patients were retrieved from the hospital record section to study the demographic variables, modes of injury, American Spinal Injury Association (ASIA) grades, salient radiological characteristics, management strategies, and the resultant clinical outcomes.

**Inclusion criteria:** Consecutive patients presenting with traumatic spinal spondyloptosis in the Department of Neurosurgery, College of Medical Sciences, Chitwan, Nepal.

**Exclusion criteria:**

• Lack of consent for participation.
• Left-against medical advice.
• Lost to follow-up, and
• Operated on in other spinal centers.

The sample size for the study was calculated by:

$$n=z^{2}\;\times \;p\;q/d^{2}=1.96\;\times \;1.96\;\times \;0.01\;\times \;0.99/0.05\times \;0.05=15.21,$$

wherein

n = minimum required sample size

z = 1.96 at 95% Confidence interval (CI)

p = reported incidence of spondyloptosis (p) at 1%

q = 1-p, and

d = margin of error at 5%.

The sample size of the study was 16 patients.

The data collected was collected and descriptive statistics were applied using the Microsoft Excel spreadsheets. The frequency distribution charts were obtained in terms of counts and percentages for each relevant variable.

Both verbal and written consent was obtained either from the patients or the next of kin for participation in the study. All their clinical and radiological data are anonymously presented in the study. The study was approved by the institutional ethical review committee of College of Medical Sciences and Teaching Hospital (IRC–COMSTH-IRC/2023-08). This study was conducted from 3rd March 2023 to 21st March 2023.

## Results

The mean age of the cohorts in our study was 40 years with an age range of 25-80 years. There was a male preponderance with a male: female ratio of 15:1. Spondyloptosis was secondary to road traffic accidents in nine (56.25%) and fall incidents in seven (43.75%). Most of the patients presented in ASIA ‘A’ neurological grade (75%) baring one (6.25%) patient in ASIA ‘B’ and three (18.75%) cases in ASIA ‘E’ neurological grades. The cervical spine was involved in the majority (68.75%) of cases. The sagittal pattern of spondyloptosis was predominant and observed in 14 patients (87.5%).

Eight (50%) patients left against medical advice after understanding the poor prognosis of the entity. two (12.5%) were managed conservatively owing to a moribund state due to pulmonary complications resulting from phrenic nerve injury and lung contusions respectively. Both of them eventually expired, six (37.5%) were operated. The posterior-only approach was undertaken in 4(66.67%) cases. Anterior only and global approach was undertaken in one (16.67%) cases each. Tracheo-oesophageal fistula occurred in two (33.33%) patients. One healed after one month of conservative management with nasogastric tube feeding. The other patient expired secondary to severe mediastinitis. Cerebrospinal fluid (CSF) leak occurred in two (33.33%) patients.

No clinical improvements were observed in patients presenting with ASIA ‘A’ neurological grades.

The overall hospital mortality was three (37.5%). The operative mortality was one (16.67%). Post-discharge, two (40%) patients eventually expired secondary to sepsis. All the surviving patients were of the ASIA ‘E’ grades.

The results of our study have been summarized in Tables 1 and 2.

**Table 1.** Anatomical levels of involvement and corresponding clinical ASIA grades.

Anatomical level of involvement	ASIA ‘A’	ASIA ‘B’	ASIA ‘E’
C2-3			2
C4-5	2		
C5-6	2		
C6-C7	2		1
C7-T1	2		
D3-4	1		
D10-11	1		
L3-4	1		
L5-S1	1	1	

**Table 2.** Demographic, radiological and clinical characteristics of the study cohort.

Study variables	Categorizations	Frequency (percentage)
Neurological presentations	ASIA ‘A’	12(75%)
	ASIA ‘B’	1(6.25%)
	ASIA ‘E’	3(18.75%)
Anatomical level of involvement	Cervical	9(56.25%)
	Thoracic	2(12.5%)
	Lumbar	1(6.25%)
	Transitional zones	4(25%)
Management strategies	Left against medical advice	8(50%)
	Conservative	2(12.5%)
	Operative	6(37.5%)
Surgical approaches	Anterior only	1(16.67%)
	Posterior only	4(66.67%)
	Global	1(16.67%)
Mortality at hospital	Surgical	1(16.67%)
	Overall	3(37.5%)

## Discussion

Most of the studies in the current literature are limited to mere case reports. ^4^ There is complete subluxation of one vertebral body over the adjacent involving all three Denis spinal columns. ^2^^–^^4^ The spinal cord is invariably damaged resulting in permanent and disabling neurological deficits among the survivors. There are also increased odds of mortality owing to the risk of associated polytrauma and complications in these cohorts. The long-term prognosis is however abysmal owing to suboptimal home-based care and liberal assess to rehabilitation facilities. ^2^ However, early stabilization promotes timely rehabilitative strategies in these cohorts. The dictum of the surgery is anatomical realignment, fixation for stability, and neural decompression. ^4^ Complete reduction may not be possible despite distraction and corpectomy. ^2^

In the largest series comprising 20 patients, the mean age of the cohorts was 27 years with a male: female ratio of 17:3. ^2^ The systematic review of cervical spondyloptosis also had a male preponderance of 70% with a mean age of 41 years. ^5^ The mean age of the cohorts in our study was 40 years and the male: female ratio was of 15:1.The most common level of involvement was at T10-L2 (55%), a mechanical transitional zone. ^2^ Ironically, there was no involvement of the cervical spine in the series, the region of maximum involvement in our study (68.5%). The study pertaining to cervical spondyloptosis had the involvement of the lower cervical spine (C6-C7 and C7-T1) in 68% of cases. ^5^ The same levels were involved in 45.45% of our study. This may be owing to the higher load with compromised mobility in the region. ^5^ All the cases presented with ASIA ‘A’ grade in the largest study and therefore were managed by short segment pedicle screw fixation in the series. ^2^ Paradoxically, three of our cases presented with ASIA ‘E’, and one patient presented with ASIA ‘B’ neurological grades. Similarly, the study relating to cervical spondyloptosis also had 21/66 (31.81%) cases presenting with ASIA ‘E’ grades. ^5^ Since we had the majority of cases with involvement of the cervical spine, anterior cervical approaches, either standalone or combined, were also adopted. Complete cord transection in observed in 35% and CSF leak in 20% of cases in the study. We had CSF leaks in 33.33% of cases. The risk of CSF leak may be prevented by ligation of the thecal sac, fibrin glue sealant, and multi-layered wound closure. ^2^

There are also high chances (40%) of clinical improvement among patients with cervical spondyloptosis. ^5^ There however increased odds of esophageal laceration and vocal cord paralysis during the surgical strategies of the same. ^5^ Tracheo-oesophageal fistula occurred in two (33.33%) patients in our study.

The reported mortality in the systematic review of cervical spondyloptosis was 11%. ^5^ The same in our study was 16.67%.

There is a continuum of physical, economic, psychological, and social burdens to both the patients and their care providers. ^2^ The impact of the disease is even more significant in middle and low-income nations. The main cause of mortality during the follow-up visits, mostly secondary to complications of bed sores, has been observed in 25% of patients. Rehabilitation plays a paramount role in the long-term outcomes in these cohorts and is the ‘bottleneck’ variable governing poor outcomes in our subcontinents. The poor economic status of the people has a ripple effect upon the same. ^2^ This is the same reason for almost 50% of our cases leaving against medical advice from our hospital.

This being a single-center study, the true incidence of the traumatic spondyloptosis may not be reflected in our single-center data. This being a rare clinical entity, a low sample size is a limiting factor of the study. Recall bias is another confounding issue.

## Conclusion

This study provides insights into the patterns of clinical presentations, radiological characteristics, management strategies, and outcome details of cohorts presenting with traumatic spinal spondyloptosis. This will help formulate management strategies and foster rational counseling. This is one of the first pilot studies to be carried out in the country relating to this rare traumatic spinal entity. This study emphasizes the implementation of a national spinal trauma data bank and the systematic implementation of dedicated neuro-rehabilitation units. This will thereby help improve the clinical outcome among these ‘socially aloof’ and marginalized subsets of neurosurgical patients.

## Data Availability

Figshare: Traumatic spinal spondyloptosis presenting in a tertiary care unit in central Nepal Item. https://doi.org/10.6084/m9.figshare.22359802.v3. ^6^ This project contains the following data:
- The data of 16 patients presenting with spondyloptosis in our center. Data are available under the terms of the Creative Commons Attribution 4.0 International license (CC-BY 4.0).
