# Traumatic spinal spondyloptosis presenting in a tertiary care unit in central Nepal

**DOI:** 10.12688/f1000research.133377.2

**Published:** 2024-05-07

**Authors:** Sunil Munakomi

**Affiliations:** 1 Neurosurgery, College of Medical Sciences, Chitwan, Nepal

**Keywords:** spondyloptosis, presentation, management, outcome

## Abstract

**Introduction:**

Traumatic spinal spondyloptosis, though rare, harbingers a high risk of mortality as well as permanent and disabling neurological deficits. They invariably become socially aloof and marginalized in most parts of our subcontinent owing to the lack of dedicated rehabilitation units amid their poor economic status. There is a paucity of studies pertaining to such rare epiphenomenon within our region.

**Materials ad Methods:**

A retrospective study of 16 patients presenting with spinal spondyloptosis in a tertiary care center in Nepal was undertaken. The clinical records of the patients were retrieved from the hospital record section to study the demographic variables, modes of injury, American Spinal Injury Association (ASIA) grades, salient radiological characteristics, management strategies, and the resultant clinical outcomes.

**Result:**

The mean age of the cohorts in our study was 40 years with an age range of 25-80 years. Most of the patients presented in ASIA ‘A’ neurological grade (75%). The cervical spine was involved in the majority (68.75%) of cases. 8 (50%) patients left against medical advice, 2 (12.5%) were managed conservatively, and 6 (37.5%) were operated. The posterior-only approach was undertaken in 4(66.67%) cases. Tracheo-oesophageal fistula occurred in 2 (33.33%) patients. And cerebrospinal fluid (CSF) leak occurred in 2 (33.33%) patients. The overall hospital mortality was 3(37.5%).

**Conclusion:**

Traumatic spinal spondyloptosis on our center mostly involved cervical spine (68.75%). 75% of the patients presented with ASIA ‘A’ neurological grade. 50% of them left against medical advice. 37.5% were operated. The overall hospital mortality was 37.5%. This study emphasizes the implementation of a national spinal trauma data bank and the systematic implementation of dedicated neuro-rehabilitation units. This will thereby help improve the clinical outcome among these ‘socially aloof’ and marginalized subsets of neurosurgical patients.

## Introduction

H.W. Meyerding first described spondyloptosis in 1938. ^1^ This is the most severe and unstable variant of translational injury that can that invariably leads to complete cord transection (above 70% cases) and ominous to permanent neurological deficits. ^2^ The survivors are compelled to be dependent on others for lifelong even for carrying out their activities of daily living (ADLs). They invariably become socially aloof and marginalized in most parts of our subcontinent owing to the lack of dedicated rehabilitation units amid their poor economic status. Since the majority of these cohorts present with poor American Spinal Injury Association (ASIA) neurological grades, the surgical dictum mostly involves anatomical fixation to assist in their early rehabilitative strategies. The combined anterior and posterior approaches are recommended only in rare circumstances for patients presenting with good ASIA neurological grades.

There is a paucity of studies pertaining to such rare epiphenomenon within our region. This study should provide insights to help frame the management algorithm among similar cohorts of patients. This will also aid in the process of patient counseling as well as foster the notion of the paramount need for dedicated neuro-rehabilitation units in our regions.

## Methods

**Figure 1.**
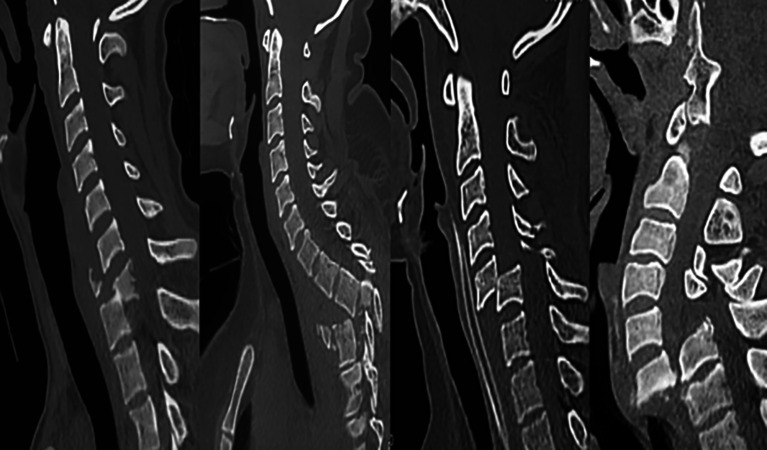
CT images of spondyloptosis at various anatomical levels of spine.

A retrospective study of consecutive cohorts of patients presenting with spinal spondyloptosis in the Emergency Department of the College of Medical Sciences was undertaken. Spondyloptosis was defined on radiological imaging (X-ray/CT/MRI) of the spine as >100% subluxation between the adjacent vertebra. The images of spondyloptosis at different levels of the spine have been demonstrated in Figure 1.

The clinical records of the patients were retrieved from the hospital record section to study the demographic variables, modes of injury, American Spinal Injury Association (ASIA) grades, salient radiological characteristics, management strategies, and the resultant clinical outcomes.

The sample size for the study was calculated by:

$$n=z^{2}\;\times \;p\;q/d^{2}=1.96\;\times \;1.96\;\times \;0.01\;\times \;0.99/0.05\times \;0.05=15.21,$$

wherein

n = minimum required sample size

z = 1.96 at 95% Confidence interval (CI)

p = reported incidence of spondyloptosis (p) at 1%

q = 1-p, and

d = margin of error at 5%.

The sample size of the study was 16 patients.

The data collected was collected and descriptive statistics were applied using the Microsoft Excel spreadsheets. The frequency distribution charts were obtained in terms of counts and percentages for each relevant variable.

All their clinical and radiological data are anonymously presented in the study. The study was approved by the institutional ethical review committee of College of Medical Sciences and Teaching Hospital (IRC–COMSTH-IRC/2023-08). This study was conducted from 3rd March 2023 to 21st March 2023.

## Results

The mean age of the cohorts in our study was 40 years with an age range of 25-80 years. There was a male preponderance with a male:female ratio of 15:1. Spondyloptosis was secondary to road traffic accidents in nine (56.25%) and fall incidents in seven (43.75%). Most of the patients presented in ASIA ‘A’ neurological grade (75%) baring one (6.25%) patient in ASIA ‘B’ and three (18.75%) cases in ASIA ‘E’ neurological grades. The cervical spine was involved in the majority (68.75%) of cases. The sagittal pattern of spondyloptosis was predominant and observed in 14 patients (87.5%).

Eight (50%) patients left against medical advice after understanding the poor prognosis of the entity. Two (12.5%) were managed conservatively owing to a moribund state due to pulmonary complications resulting from phrenic nerve injury and lung contusions respectively. Both of them eventually expired, six (37.5%) were operated. The posterior-only approach was undertaken in 4(66.67%) cases. Anterior only and global approach was undertaken in one (16.67%) cases each. Tracheo-oesophageal fistula occurred in two (33.33%) patients. One healed after one month of conservative management with nasogastric tube feeding. The other patient expired secondary to severe mediastinitis. Cerebrospinal fluid (CSF) leak occurred in two (33.33%) patients.

No clinical improvements were observed in patients presenting with ASIA ‘A’ neurological grades.

The overall hospital mortality was three (37.5%). The operative mortality was one (16.67%). Post-discharge, two (40%) patients eventually expired secondary to sepsis. The results of our study have been summarized in Tables 1 and 2.

**Table 1.** Anatomical levels of involvement and corresponding clinical ASIA grades.

Anatomical level of involvement	ASIA ‘A’	ASIA ‘B’	ASIA ‘E’
C2-3			2
C4-5	2		
C5-6	2		
C6-C7	2		1
C7-T1	2		
D3-4	1		
D10-11	1		
L3-4	1		
L5-S1	1	1	

**Table 2.** Demographic, radiological and clinical characteristics of the study cohort.

Study variables	Categorizations	Frequency (percentage)
Neurological presentations	ASIA ‘A’	12(75%)
	ASIA ‘B’	1(6.25%)
	ASIA ‘E’	3(18.75%)
Anatomical level of involvement	Cervical	9(56.25%)
	Thoracic	2(12.5%)
	Lumbar	1(6.25%)
	Transitional zones	4(25%)
Management strategies	Left against medical advice	8(50%)
	Conservative	2(12.5%)
	Operative	6(37.5%)
Surgical approaches	Anterior only	1(16.67%)
	Posterior only	4(66.67%)
	Global	1(16.67%)
Mortality at hospital	Surgical	1(16.67%)
	Overall	3(37.5%)

## Discussion

A concurrent shearing and axial compression vector disrupts all three Denis columns in spondyloptosis. ^3^^,^^4^ The narrower cord to canal ratio and the tenuous blood supply also increases odds to neurological deficits, mostly in thoracic variant. ^5^ Complete neurological deficit (ASIA’A’) is observed in almost 80% of cases. ^6^ Saving (free-floating) fractures of the vertebral arch, described by Bohler in 1948, can sometimes enigmatically spare the spinal cord owing to spontaneous decompression. ^4^^,^^5^

Motor vehicle accidents and substantial falls mostly accounts for traumatic form of spondyloptosis. ^5^ This can be anterior (almost 80%), posterior, or lateral relative to the caudal vertebra depending upon the direct of the impact, as described by Denis and Burkus. ^7^ This mostly often occurs in the transitional spinal regions (C7-T1, T10-L2, L4-S1 and L4/L5). ^1^^,^^7^ Furthermore there is least resistance along the first sacral vertebral foramen. ^6^

Cervical spondyloptosis harbinger risk of injuries to the phrenic nerve and vertebral artery. ^2^ There is risk of aortic, vena cava, and iliac injuries in thoracic and lumbar variants. ^3^ Concomitant chest complications, such as rib fractures, pneumothorax, and hemothorax can occur in thoracic spondyloptosis. ^5^ It is also essential to rule out fractures at non-contiguous spinal regions that can easily get overlooked. ^3^

There are currently no clear guidelines for the management of traumatic spondyloptosis. ^1^ Distraction, unlocking, anatomic fixation, and arthrodesis are the dictum of surgical management. ^6^ The core principle of care is restoration and stabilization of spinal alignment to facilitate early mobilization and rehabilitation in the patient. ^2^ The risks/benefits ratio governs a significant interplay the management algorithm. ^8^ Combined 360 degrees and 540 degrees approaches may be justified for circumferential and robust fixation among cohorts with preserved neurology.

Traction is first applied to provide stability, minimize pain, and to restore the spinal alignment, especially in cervical spondyloptosis. ^5^^,^^7^ The traction is applied 2 cm posterior to the inter-aural line to facilitating flexion. An initial 4 kg followed by gradual increment by 4 kg increments every 20 minutes (max 63 kg) until complete reduction or neurological deterioration (whichever comes earliest) is seen. The posterior variant resulting from the disruption of the soft tissue stabilizers have a high chance of complete reduction following traction. ^6^

Distraction and unlocking may facilitate intra-operative reduction. ^6^ A combined anterior and posterior approach fusion, with intra-operative neuro-monitoring is advocated for patients with preserved neurology. ^8^ Intraoperative axilla-pelvic distraction followed by corpectomy, or spondylectomy with cage placement have been described for thoracic spondyloptosis. ^9^ Such spinal shortening procedure poses significant risk of injuries to aorta or vena cava during distraction. ^9^ Similarly, Corpectomy and cage placement at L5-S1 is technically demanding owing to its inherent lordosis. ^3^ Grob’s, Gaines and Nichols methods have significant surgical risks. ^1^ Furthermore, pelvic instrumentation may be justified to prevent pseudo-arthrosis. ^1^ Therefore standalone long-segment posterior instrumentation is often the ‘work-horse’ in the surgical management of thoracic and lumbar spondyloptosis, especially in ASIA ‘A’ and ‘B’ subtypes’, to facilitate their rehabilitative strategies. ^3^ Standalone anterior or posterior approaches may be justified in cervical spondyloptosis with similar neurological status depending upon the reducibility of the subluxation after traction.

In the largest series comprising 20 patients, the mean age of the cohorts was 27 years with a male: female ratio of 5.6:1. ^10^ The systematic review of cervical spondyloptosis also had a male preponderance of 70% with a mean age of 41 years. ^11^ In a most recent study comprising 17 patients, the mean age was 34.5 years, male to female ratio of 2.4:1. ^7^ The mean age of the cohorts in our study was 40 years and the male: female ratio was of 15:1.The most common level of involvement was at T10-L2 (55%), a mechanical transitional zone. ^10^ Ironically, there was no involvement of the cervical spine in this study, the region of maximum involvement in our study (68.5%). The study pertaining to cervical spondyloptosis had the involvement of the lower cervical spine (C6-C7 and C7-T1) in 68% of cases. ^11^ The same levels were involved in 45.45% of our study. This may be owing to the higher load with compromised mobility in the region. ^5^ All the cases presented with ASIA ‘A’ grade in the largest study and therefore were managed by short segment pedicle screw fixation. ^10^ Three of our cases presented with ASIA ‘E’, and one patient presented with ASIA ‘B’ neurological grades. Similarly, the study relating to cervical spondyloptosis also had 21/66 (31.81%) cases presenting with ASIA ‘E’ grades. ^11^ Since we had the majority of cases with involvement of the cervical spine, anterior cervical approaches, either standalone or combined, were also adopted. We had CSF leaks in 33.33% of operated cases. The risk of CSF leak may be prevented by ligation of the thecal sac, fibrin glue sealant, and multi-layered wound closure. ^10^ There is also increased odds of esophageal laceration and vocal cord paralysis during the surgical strategies of the cervical spondyloptosis. ^11^ Tracheo-oesophageal fistula occurred in two (33.33%) patients in our study.

The reported mortality in the systematic review of cervical spondyloptosis was 11%. ^11^ Two mortalities occurred in the most recent study secondary to pulmonary complications and DVT. ^7^ The same in our study was 16.67%.

Studies have shown no significant differences between the standalone anterior or posterior approaches with combined approaches with regards to improvement in neurology. ^7^ Similarly, no association has been found between injury to surgery time and outcome. ^7^ However, duration of injury to surgical time was significantly associated with postoperative residual listhesis. ^7^ Complete reduction may not be possible despite distraction and corpectomy. ^10^ Posterior alone approach was used in 93.3% of cases in one series. ^7^

Multidisciplinary care is prudent for optimal outcomes. ^9^ The overall prognosis is poor, and this has a continuum and multispectral debilitating impact upon the patient’s quality of life. ^1^ Bleak outcomes with devastating long-term physical disability and psychosocial sequelae. 88.2% showed no improvement in one study. ^7^ The long-term prognosis is however abysmal owing to suboptimal home-based care and liberal assess to rehabilitation facilities especially in low income nations. ^10^ The main cause of mortality during the follow-up visits, mostly secondary to complications of bed sores, has been observed in 25% of patients. Lack of dedicated rehabilitation is the ‘bottleneck’ variable governing poor outcomes in our subcontinents. The poor economic status of the people has a ripple effect upon the same. ^10^ This is the same reason for almost 50% of our cases leaving against medical advice from our hospital.

Despite being a rare clinical entity, a low sample size is a limiting factor of the study. The true incidence of the traumatic spondyloptosis in our region may not be reflected for our single-center data.

## Conclusion

Traumatic spinal spondyloptosis on our center mostly involved cervical spine (68.75%). 75% of the patients presented with ASIA ‘A’ neurological grade. 50% of them left against medical advice. 37.5% were operated. The overall hospital mortality was 37.5%. This study provides insights into the patterns of clinical presentations, radiological characteristics, management strategies, and outcome details of cohorts presenting with traumatic spinal spondyloptosis. This will help formulate management strategies and foster rational counseling. This is one of the first pilot studies to be carried out in the country relating to this rare traumatic spinal entity. This study emphasizes the implementation of a national spinal trauma data bank and the systematic implementation of dedicated neuro-rehabilitation units. This will thereby help improve the clinical outcome among these ‘socially aloof’ and marginalized subsets of neurosurgical patients.

## Data availability

Figshare: Traumatic spinal spondyloptosis presenting in a tertiary care unit in central Nepal Item. https://doi.org/10.6084/m9.figshare.22359802.v3. ^12^

This project contains the following data:

• The data of 16 patients presenting with spondyloptosis in our center.

Data are available under the terms of the Creative Commons Attribution 4.0 International license (CC-BY 4.0).
