# Supplementary material for: Traumatic spinal spondyloptosis presenting in a tertiary care unit in central Nepal
Source: F1000Res. 2025 Jun 2;12:474. Originally published 2023 May 9. [Version 3] doi: 10.12688/f1000research.133377.3 (PMC13254520; doi:10.12688/f1000research.133377.3)
Supplement: Peer review history

# Reviewer report: Reviewer response for version 3

**DOI:** 10.5256/f1000research.183026.r486487

**Published:** 2026-06-10

**Authors:** Amit Thapa

**Affiliations:** 1 Kathmandu Medical College Teaching Hospital, Kathmandu, Nepal

The article is an observational, retrospective study detailing the clinical presentations, radiological characteristics, surgical management, and outcomes of 16 patients presenting with traumatic spinal spondyloptosis (severe spinal subluxation >100%) at a single tertiary care center in Chitwan, Nepal.

 While the topic is clinically relevant and provides rare, valuable data from a developing country, the current format contains contradictory methodology, flawed statistical reasoning, and scientific "spinning" that severely undermines its credibility. The paper requires major revision

- Mention the type of the study in the manuscript as retrospective based on records rather than “An observational study of consecutive cohorts of patients”.
- Do not mention duration of study as between “3rd March 2023 to 21st March 2023”, rather say the duration when the patients were included in the study and how long were they followed up
- Because this is a retrospective case series/chart review of an extremely rare condition over a fixed chronological window, a prospective sample size calculation is scientifically inappropriate and forced. Please remove this calculation and simply state that "all consecutive cases presenting to the center within the study period were included."
- In the results and discussion, ensure there is absolutely no ambiguity regarding neurological improvement. To prevent dangerous clinical misinterpretation, provide a clear, itemized Master Table matching each individual patient's serial number to their admission ASIA score, injury level, management type (surgical vs. conservative), and final outcome (ASIA score on fup)/mortality.
- expand the discussion to address the specific biomechanics, mechanisms of injury (e.g., specific types of falls from heights or unique road traffic patterns in central Nepal), or pre-hospital transport factors that explain this unique cervical dominance and high survival rate to the ER
- modify the conclusion to acknowledge the massive loss-to-follow-up (50% LAMA rate) as a major limitation, and discuss how the lack of regional neuro-rehabilitation infrastructure directly compromises the natural history of these patients.

Is the work clearly and accurately presented and does it cite the current literature?

Partly

If applicable, is the statistical analysis and its interpretation appropriate?

No

Are all the source data underlying the results available to ensure full reproducibility?

Partly

Is the study design appropriate and is the work technically sound?

No

Are the conclusions drawn adequately supported by the results?

Partly

Are sufficient details of methods and analysis provided to allow replication by others?

Partly

Reviewer Expertise:

Spine, peripheral nerves and brain

I confirm that I have read this submission and believe that I have an appropriate level of expertise to confirm that it is of an acceptable scientific standard, however I have significant reservations, as outlined above.

# Reviewer report: Reviewer response for version 2

**DOI:** 10.5256/f1000research.165775.r382973

**Published:** 2025-05-20

**Authors:** Pratyush Shrestha

**Affiliations:** 1 National Institute of Neurological and Allied Sciences, Bansbari, Nepal

1) Why has sample size calculation been done here? This is not a prospective study, readers concentration is misdirected

 2) The study reports different patients with different ASIA grades; and different patient receiving different forms of treatment. It would be worthful if the author mentioned which specific patient received which treatment modality. The 3 patients with ASIA E status, how they fared is something every reader would be interested to know.

 3) The study concludes on neurorehabilitation and also in the introduction mentions these patients being socially aloof and marginalized; which is not a finding of this study. If the author could have followed these patients in a point in time later, how they fared after the accident, these conclusions would be of value. How long these patients lived, what happened after they left against medical advice would be a great addition on the message the study gives, i.e, the need of a national registry which has rightly been stressed by the author.

Is the work clearly and accurately presented and does it cite the current literature?

Yes

If applicable, is the statistical analysis and its interpretation appropriate?

Not applicable

Are all the source data underlying the results available to ensure full reproducibility?

Yes

Is the study design appropriate and is the work technically sound?

Yes

Are the conclusions drawn adequately supported by the results?

Partly

Are sufficient details of methods and analysis provided to allow replication by others?

Yes

Reviewer Expertise:

spine and brain

I confirm that I have read this submission and believe that I have an appropriate level of expertise to confirm that it is of an acceptable scientific standard, however I have significant reservations, as outlined above.

# Reviewer report: Reviewer response for version 2

**DOI:** 10.5256/f1000research.165775.r276092

**Published:** 2024-06-07

**Authors:** Ibrahim Dao

**Affiliations:** 1 Nazi Boni University, Bobo Dioulasso, Burkina Faso

Dear editor

 The author deal with an interesting topic of 16 traumatic spinal spondyloptosis managed in their department including 11 cases of cervical spine. 

 Despite surgical management in some cases, there was any improvement in patient with ASIA A at presentation.

 Comments to authors:

 1. For a better evaluation of the preoperative lesion above all in cervical spine, did you perform MRI.

 If yes, you should add some MRI images.

 If no, explain.

 2. Post operative control imaging is very important. Please provides them. 

 3. Please your keywords did not cope properly with the manuscript. This will make its the research difficult. after Indexing

Is the work clearly and accurately presented and does it cite the current literature?

Yes

If applicable, is the statistical analysis and its interpretation appropriate?

Yes

Are all the source data underlying the results available to ensure full reproducibility?

Yes

Is the study design appropriate and is the work technically sound?

Yes

Are the conclusions drawn adequately supported by the results?

Yes

Are sufficient details of methods and analysis provided to allow replication by others?

Yes

Reviewer Expertise:

Neurosciences

I confirm that I have read this submission and believe that I have an appropriate level of expertise to confirm that it is of an acceptable scientific standard.

# Reviewer report: Reviewer response for version 1

**DOI:** 10.5256/f1000research.146362.r172993

**Published:** 2023-12-20

**Authors:** Bidur KC

**Affiliations:** 1 Department of Neurosurgery, Kathmandu Medical College and Teaching Hospital, Kathmandu, Nepal

1. In the material and methods of abstract section, type of study should be mentioned.
2. In the result part of abstract section, last paragraph all the surviving patients were of ASIA grade E should be omitted, because this sentence gives a false sense of good prognosis after treatment in patients with spondyloptosis.
3. Conclusion part of abstract section, it is not according to the objective of the study and results of the study.
4. In method section of manuscript, retrospective type of study should be mentioned. Looks like there is a methodological flaw because ethical review committee has approved the study in 2023, sample was taken from January 2017 and in methodology it was written that data were taken from the hospital record section and later part it was mentioned that author has taken verbal and written consent.
5. In the result section of manuscript, as exclusion criteria has mentioned to exclude those patients who went on leave against medical advice but in the data, 8 patient who left against medical advice were also included. So actual study of remaining 8 patients should have been included in the results to match the objective of the study. In the last paragraph instead of writing all surviving patients were of ASIA E grades, mention the exact number of patients because this sentence giving a false sense of good prognosis after treatment in patients with spondyloptosis.
6. Discussion part of manuscript is slightly short, should have been elaborative.
7. Conclusion should be drawn according to the results of manuscript. Must be rewritten.
8. Too few number of references included. If the author make the discussion part elaborative then references will also increase in number.
9. This study is retrospective in nature with only 8 patients available for analysis of final results.

Is the work clearly and accurately presented and does it cite the current literature?

Partly

If applicable, is the statistical analysis and its interpretation appropriate?

Not applicable

Are all the source data underlying the results available to ensure full reproducibility?

Yes

Is the study design appropriate and is the work technically sound?

No

Are the conclusions drawn adequately supported by the results?

No

Are sufficient details of methods and analysis provided to allow replication by others?

Partly

Reviewer Expertise:

Neurosurgery

I confirm that I have read this submission and believe that I have an appropriate level of expertise to state that I do not consider it to be of an acceptable scientific standard, for reasons outlined above.
